# High Hydrostatic Pressure Treatment of Oysters (*Crassostrea gigas*)—Impact on Physicochemical Properties, Texture Parameters, and Volatile Flavor Compounds

**DOI:** 10.3390/molecules26195731

**Published:** 2021-09-22

**Authors:** Yuyang Ma, Runfang Wang, Tietao Zhang, Yunsheng Xu, Suisui Jiang, Yuanhui Zhao

**Affiliations:** 1 College of Food Science and Engineering, Ocean University of China, Qingdao 266003, China; myuyang@yeah.net (Y.M.); wrf0814@126.com (R.W.); jiangsuisui713@163.com (S.J.); 2 College of Food Science and Engineering, Hainan Tropical Ocean University, Sanya 572022, China; ttzhang1973@163.com (T.Z.); lyxys@hntou.edu.cn (Y.X.)

**Keywords:** oyster, high hydrostatic pressure treatment, fatty acid composition, texture parameters, volatile flavor, edible quality

## Abstract

High hydrostatic pressure (HHP) treatment is a non-thermal processing technology, which is widely used in the food processing field at present. In this study, the effects of HHP treatment (100~500 MPa for 5 min) on the physicochemical properties, texture parameters, and volatile flavor compounds of oysters were investigated. The results showed that HHP treatment increased the water content while reducing the crude protein and ash content of the oyster. Texture parameters showed that HHP treatment improved the hardness, springiness, chewiness, and cohesiveness of oysters, compared with the control group. In addition, the saturated fatty acid (SFA) content was slightly increased after HHP treatment, while the difference in monounsaturated fatty acid (MUFA) and polyunsaturated fatty acid (PUFA) content was not significant. Furthermore, HHP increased hexenoic aldehyde, 2,4-heptadienal, 1-octene-3-ol, and 2-octen-1-ol and decreased the contents of 3. 6-nadien-1-ol, 3-octanone, and 2-undecanone, suggesting that HHP might inhibit the fishiness of oyster and showed a positive effect on its flavor. Based on the above results, HHP improved the edible qualities such as texture properties and volatile flavor of oysters. This meets the requirements of consumers on the edible quality of seafood and provides new ideas for the development of seafood.

## 1. Introduction

Aquatic food is rich in nutritional value, widely consumed in the world, and has great consumption potential [1,2]. Oyster (*Crassostrea gigas*) is a kind of nutritious food, which constitutes a necessary ingredient for a healthy diet [3,4]. The freshness and hygiene index of the oyster has reached the international level, and it can be eaten directly as raw food [5]. In addition, they can also be consumed by steaming, boiling, grilling, frying, and canning foods in China and some Southeast Asian countries [6,7]. However, when oysters grow in seawater containing pathogens, an infection may occur, which may lead to various food-borne diseases caused by eating oysters [8]. Every year, many diseases such as diarrhea and primary septicemia caused by eating raw oysters are reported around the world [9,10]. Oysters are safe after heat treatment as compared to raw oysters. However, due to the change of approximate composition and fatty acid composition, there may be significant differences between hot-processed oysters and raw oysters in terms of eating quality, including flavor, color, smell, texture, and nutritional value [5,11].

High hydrostatic pressure (HHP) treatment is a non-thermal processing technology that has attracted widespread attention in the field of food processing and preservation [12,13,14,15]. Previous studies have shown that HHP treatment could improve microbial safety and extend the shelf life of food while having minimal impact on sensory, physicochemical properties, flavor, and nutritional characteristics [16,17,18]. In addition, HHP technology is increasingly applied to oysters [7,19]. It has been reported that the total microbial count in oysters was significantly reduced after HHP treatment, and the microbial count remained at a reduced level in subsequent preservations, which prolonged the shelf life of oysters [20]. After HHP treatment, the *Vibrio vulnificus* and other pathogenic microorganisms in oysters were eliminated [21,22]. Moreover, HHP treatment had less effect on the changes of fatty acids and non-volatile flavor active substances in oysters compared with raw oysters [7]. However, the research on HHP on oysters mainly focuses on its effects on microorganisms, physical and chemical properties, storage stability, and non-volatile flavor active compounds.

Besides nutrition and freshness, texture characteristics and volatile flavor are also important factors for consumers’ acceptability of seafood products [23]. The formation of volatile flavor in aquatic products is related to the chemical reaction of lipids, proteins, and saccharides [24]. It is well known that HPP significantly affects the above chemical reactions. Additionally, compared with mammals, the solubility of the meat of aquatic products is higher, which causes extensive softening of the meat and reduces the edible quality of aquatic products [25]. Therefore, the meat quality of aquatic products is usually required to have certain hardness and cohesion when being consumed. Similarly, HPP has been reported to alter the protein structure and further affect the texture properties of aquatic products [26]. Based on the above, we speculate that HHP treatment may have a significant impact on texture characteristics and volatile flavor components of oysters. However, there are few reports on texture characteristics and volatile odor components of oysters treated by HHP. Therefore, this study explored the effect of HHP on the edible quality of oysters by combining texture parameters, fatty acid spectrum, electronic nose, and volatile component analysis. The current research will provide a new reference for the HHP processing of oysters and reveal the great application potential of HHP treatment as a new processing technology.

## 2. Results and Discussion

### 2.1. Effect of HHP Treatment on Proximate Compositions and pH

The proximate composition changes after different pressure treatments are shown in Table 1. After HHP treatment, the moisture content of oysters increased from 82.45% (0.1 MPa) to 84.71% (500 MPa). It was speculated that the increase in water content might be due to HHP increasing the hydration of protein, which directly caused the increase in water absorption of protein. The protein content decreased from 12.14% (0.1 MPa) to 10.52% (500 MPa). Furthermore, the crude fat content and glycogen content did not change significantly. Meanwhile, the ash content was slightly reduced, which might be related to the leaching of oyster components by the interlayer fluid [27]. In the present study, the increase of water content may dilute other components in oysters. This is consistent with the decrease of crude protein and ash content in oysters after HHP treatment.

The pH value of aquatic products is an important indicator to evaluate the quality of food [28]. As shown in Table 1, the pH of all samples was higher than 6.0 compared with the control group. When HHP treatment was less than 300 MPa, the pH value of oysters increased slightly. However, the pH of oysters increased after HHP treatment pressures above 300 MPa, but there was no difference between the treatment groups (300–500 MPa). The increase in pH might be attributed to the depolymerization of certain proteins and the exposure of certain polar groups under high pressure [11].

### 2.2. Effect of HHP Treatment on Texture Profile

Hardness is an internal binding force for food to maintain its own shape [23]. As shown in Table 2, when the HHP pressure was higher than 300 MPa, the hardness of the oysters was significantly increased (*p* < 0.05). HHP treatment has been reported to cause degeneration and deformation of myofibrillar protein. With the increase of pressure, the morphology of myofibrillar protein usually changes from tight aggregation to dense uniformity [29]. During this process, the pressure of HHP can cause non-covalent bond changes, thus affecting the hardness of oysters [30]. Moreover, HHP may also promote the formation of intermolecular disulfide bonds and produce some high molecular polypeptides, resulting in increased hardness [31].

Springiness reflects the degree of recovery of the oyster after being deformed by an external force, and the springiness of oyster closed muscles is positively related to the binding force between muscles [32]. Table 2 shows that the springiness change of the sample is not significant compared with the control sample at the low pressure (<300 MPa), while as the pressure continues to increase (300–500 MPa), the springiness increases significantly (*p* < 0.05). Springiness has been reported to be closely related to sarcoplasmic proteins. Sarcoplasmic proteins form insoluble precipitates at a pressure above 140 MPa. In addition, when the mass concentration of sarcoplasmic protein was higher than 50 mg/mL, the protein formed a gel, and the strength of the gel increased with the increase of pressure, which directly led to the increase of springiness [33]. Furthermore, the changes in springiness might also be related to changes in myofibrils during gel formation [34].

Chewiness represents the tenderness of food. The effect of HHP treatment on the chewiness of oysters is shown in Table 2. After HHP treatment, the chewing ability of oysters was not significantly different from the control group between 100 and 300 MPa (*p* > 0.05). However, as the pressure increased to 400 MPa, the chewiness of oysters increased significantly (*p* < 0.05). It was reported that the chewiness of chicken breast was improved after high-pressure treatment (300–600 MPa), which is similar to the present results [35]. In this experiment, the effect of HHP treatment on the chewability and hardness of oysters was similar, which is consistent with the previous conclusion that the hardness and chewability were positively correlated [36].

Furthermore, the cohesiveness of oysters increased significantly (*p* < 0.05) when the pressure was greater than 300 MPa (Table 2). Hardness, springiness, cohesiveness, and chewiness reflect the degree of muscle softness, the ability to resist external force recovery, the tightness of muscle tissue bonding, and “bite strength” [37]. In general, the structural changes of contractile proteins in myofibrillar proteins in muscle were the main factors leading to changes in texture [38]. Our previous report showed that after HHP treatment, the unfolding of the protein and the extension of the peptide segment exposed some internal groups, such as hydrophobic groups and SH inter-group, which led to an increase in the mercapto group. The hydrophobic groups in the protein enhanced the hydration of protein, while the increase in the mercapto group content improved the gel properties of the protein, all of which affected the hardness, springiness, chewiness, and cohesiveness of the oyster [39]. The above results indicated that HHP treatment significantly changed the texture characteristics of oysters.

### 2.3. Effect of HHP Treatment on Fatty Acid Composition

Marine lipids contain large amounts of long-chain triglycerides with high levels of unsaturated fatty acids, as well as high levels of unsaturated phospholipids, which are usually closely related to total lipid levels [40]. In addition, previous studies have reported that lipids are susceptible to external influences during different processing processes, such as pressure, heat, and other processes, thereby affecting the biological components [41]. Therefore, we measured the fatty acid profiles under different pressure treatment conditions, and the results are shown in Table 3. After HHP treatment, the composition of fatty acids changed slightly. A total of 25 fatty acids were detected, including 11 saturated fatty acids (SFA), 6 monounsaturated fatty acids (MUFA), and 8 polyunsaturated fatty acids (PUFA). Among them, C16:0 was the main component of fatty acids, accounting for 23.23~25.33%, followed by C18:0 (11.38–12.58%), C20:5n3 (5.35–6.64%), and C22:6n3 (6.10–7.68%). Compared with the control group, the saturated fatty acid (SFA) was slightly increased in the HHP group, while the monounsaturated fatty acid (MUFA) and polyunsaturated fatty acid (PUFA) were almost unchanged. It is worth noting that after HHP treatment, the content of n-3 PUFA (mainly C20:5n3 and C22:6n3) in the HHP treatment group was significantly higher than that in the control group (*p* < 0.05). Therefore, the ratio of n-3 PUFA to n-6 PUFA in the HHP treatment group was significantly higher than that in the control group (*p* < 0.05). The levels of long-chain n-3 and n-6 fatty acids (commonly called PUFAs) and their ratios (n-3/n-6) are considered important to human health [42].

### 2.4. Electronic Nose Result Analysis

**Figure 1:**
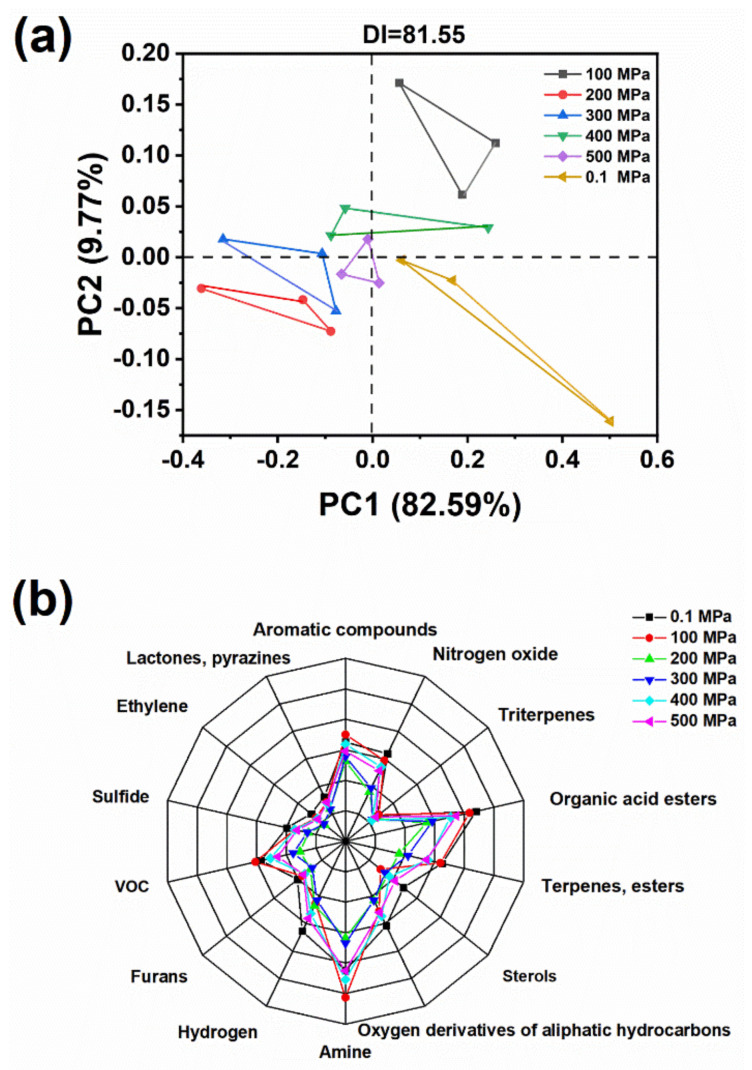
Principal component analysis of electronic nose (**a**) and flavor radar map (**b**).

Principal component analysis (PCA) is a method used to determine the similarity between objects in a two-way data set (samples of sensors). The DI value indicates the degree of separation between samples. As shown in Figure 1a, the degree of separation between different sample groups was 81.55, indicating that the flavor of oyster samples after different pressure treatments had changed significantly. Moreover, the contribution rates of the first main component (first main axis) and the second main component (second main axis) were 82.59% and 9.77%, respectively, and the total contribution rate was 92.36%. The distribution of the sample on the PCA graph showed certain regularity; the samples were divided into four areas on the PCA graph. The control group was distributed in a separate area, 100 MPa-treated samples were distributed in a single area, 200- and 300 MPa-processed samples were distributed in a single area, and 400 and 500 MPa-processed samples were distributed in a separate area. The distribution of the four samples treated above 200 MPa was relatively close, indicating that their similarities were higher. Therefore, these results demonstrated that the samples treated with high pressure had a significant difference in odor compared with the control sample. As shown in Figure 1b, there were differences in the response values of each sensor in the samples. The control sample (0.1 MPa) had the highest response to organic acid esters, followed by amine, aromatic compounds, nitrogen oxides, terpenes and esters, and hydrogen. Lactones and pyrazines, and ethylene had the lowest response values. After HHP treatment, the response values of most sensors declined to varying degrees.

### 2.5. Effect of HHP Treatment on Volatile Compounds

The GC–MS elution peak was searched through the database NIST0 8.L spectral library, and the composition and concentration of volatile components during different HHP treatments were further obtained through quantitative analysis. Twenty-nine volatile compounds were detected by HS–SPME/GC–MS, including seven aldehydes, five alcohols, four esters, two ketones, seven hydrocarbons, and four other classes of compounds (Table 4). A total of 19 volatile components were detected in raw oysters, and 15, 15, 19, 20, and 24 volatile components were detected at 100, 200, 300, 400, and 500 MPa, respectively. These volatile components mainly include aldehydes, alcohols, hydrocarbons, ketones, and esters. When the pressure was higher than 300 MPa, the number of volatile components in the HHP treatment samples was significantly higher than that in the control group.

**Figure 2:**
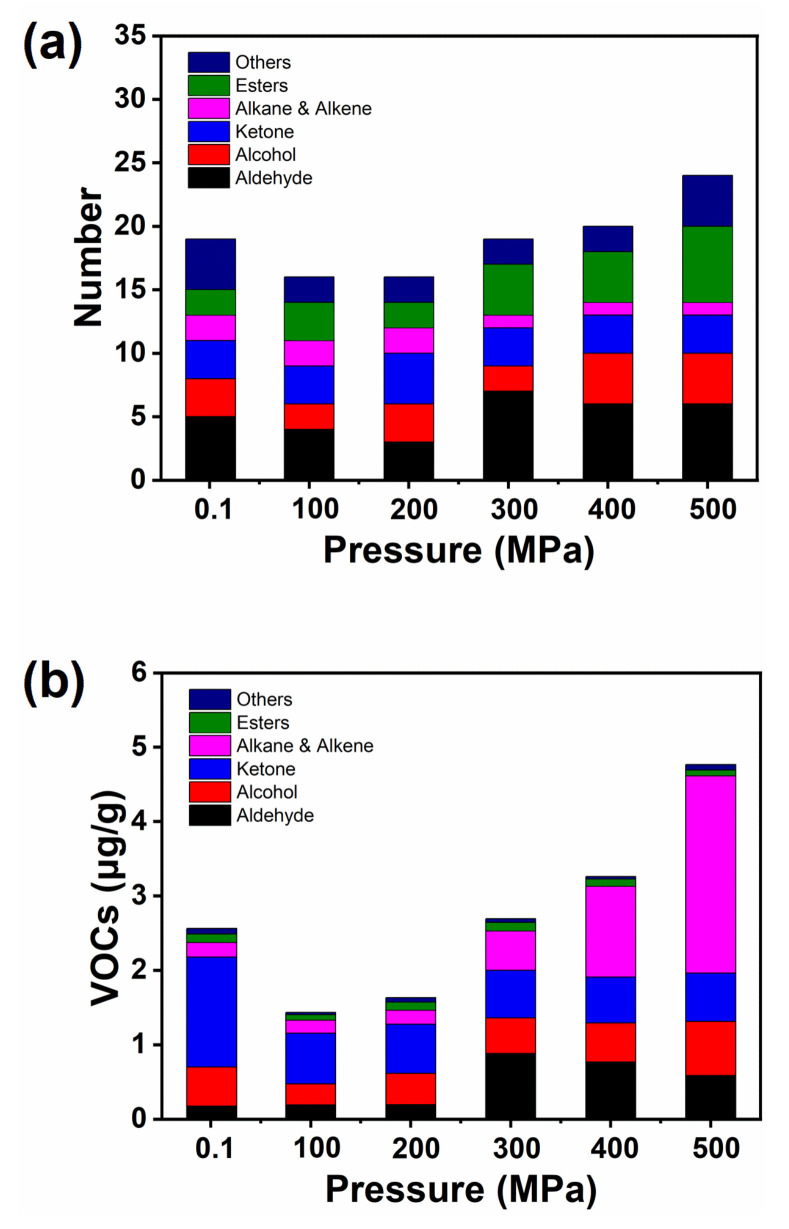
Change of the number (**a**) and total content (**b**) of volatile flavor components in oysters treated with different HHP.

The amount and relative contents of volatile components of HHP-treated oysters and the control group are shown in Figure 2. The detected volatile components include aldehydes, alcohols, ketones, esters, and hydrocarbons. After HHP treatment, the amounts of esters, aldehydes, and alcohols increased, while the number of hydrocarbons decreased, and ketones did not change (Figure 2a), indicating that HHP treatment changed the composition of oysters’ volatile components. Compared with untreated oysters, the relative content of aldehydes, alcohols, and hydrocarbons increased significantly after HHP treatment, the content of ketones decreased, and there was no change in esters (Figure 2b).

Aldehydes play an important role in the flavor of oysters [43]. In the highest amount in raw oyster was 2,6-nonadienal, followed by 2-nonenal, 2,4-heptadienal, 2,4-heptadienal, and octanal. In general, HHP treatment affects the main aldehyde volatile compounds. After HHP treatment, the 2,6-nonadienal, 2-nonenal, 2,4-heptadienal, and octanal in oysters increased significantly. In addition, when the pressure was higher than 300 MPa, new volatile components (hexenal and 2,4-octadiene aldehyde) were produced, compared with the control group. It is generally believed that aldehydes are generated by oxidative degradation of polyunsaturated fatty acids in oysters [44]. Hexenoic aldehyde and 2,4-heptadienal had a grassy fragrance; heptanal and benzaldehyde had a pleasant nutty aroma [45]. After HHP treatment, the concentration and number of aldehyde compounds were higher than those in the control group, which might enhance the pleasant smell of oysters.

Alcohol compounds have a higher odor threshold, so they contribute less to flavor unless the concentration is high or unsaturated alcohol [46]. A total of five alcohols have been detected, including 2-penten-1-ol, 1-octen-3-ol, 2-octen-1-ol, (6Z)-nonen-1-ol, and 3,6-nonadien-1-ol. After HHP treatment, 1-octen-3-ol increased compared with the control group, while 3,6-nonadien-1-ol concentration decreased. In addition, HHP treatment produced two volatile compounds, 2-penten-1-ol and 2-octen-1-ol, and 6Z-nonyl-1-ol disappears. 1-octene-3-ol was unsaturated alcohol with a relatively low threshold, with mushroom aroma being one of the main volatile alcohols in oysters [47]. 3,6-nonadien-1-ol has a fishy smell, and the decrease in its content indicates that the fishy components in the oyster were reduced after HHP treatment. 2-penten-1-ol and 2-octen-1-ol had a plant flavor, and they had a positive effect on the flavor of oysters [44].

Compared to alcohols, ketones have a lower threshold. Microbial, lipid oxidation, or amino acid degradation may produce volatile ketones [43]. In this study, a total of 2 aldehydes were detected, including 3-octanone and 2-undecanone. Compared with the untreated oysters, HHP treatment reduced the concentration of 3-octanone and 2-undecanone. When the pressure exceeds 300 MPa, the 2-undecanone even disappears. 3-octanone was considered to be the main volatile compound in mushrooms, producing a mushroom-like aroma; 2-undecanone had a fatty smell [47]. Ketones were considered to be an off-flavors produced by long-term storage [44]. Therefore, the effect of HHP treatment on volatile aldehydes showed that HHP treatment could control off-flavors generation.

The highest content of volatile substances in the detected oyster samples are hydrocarbons. However, due to the higher odor threshold of hydrocarbons, they have little effect on the overall flavor of oysters [48].

## 3. Materials and Methods

### 3.1. Oyster Materials

Adult oysters (*Crassostrea gigas*) were purchased from the aquatic product market near Ocean University of China in December 2019. All oyster samples were processed experimentally within 24 h after harvest. Then, 48 oysters (135.63 ± 16.00 mm of shell height, 151.38 ± 13.26 g of wet weight) with full gonads were selected for subsequent experiments. They were all washed with purified water, put into polyethylene plastic bags, and then vacuum-heat-sealed.

### 3.2. HHP Treatment of Oysters

The prepared oysters were shelled and placed in an HHP treatment device (CQC2-600, Beijing Suyuan Zhong Tian Co, Ltd., China), with water as the pressurized medium. Single-cycle treatments with different pressure, at 100, 200, 300, 400, and 500 MPa, were applied to oyster samples, the holding time was 5 min, and 0.1 MPa was used as the control group. The speed of pressure regulation was 100 MPa/min, and the time for maintaining the pressure was 5 min. Subsequently, one part was used for the determination of texture, and the other part was stored at −80 °C for the determination of biochemical and volatile components. Each HHP treatment condition was completed in three separate runs.

### 3.3. Proximate Composition Analysis

Moisture was determined by drying (at 60 °C) to constant weight. The crude protein content was determined by using a fully automatic Kjeldahl analyzer (FOSS-Soxtec 2050, Sweden). Crude fat was extracted through a Soxhlet extractor, according to previous reports [49]. Glycogen content was determined by using a glycogen content kit (Jiancheng Biological Engineering Institute, Nanjing, China). The ash content was determined by gravimetric method, and the sample was placed at a temperature of 550 °C and incinerated for 24 h. All samples were in triplicate.

### 3.4. Changes in pH

The oyster sample was homogenized with deionized water. The pH value of oysters after HHP treatment was measured with a pH meter (M0104; Mettler Toledo, Switzerland).

### 3.5. Fatty Acid Analysis

Fatty acids were determined according to previously reported methods, which were slightly modified [7]. Briefly, fatty acid methyl esters (FAMEs) were prepared by transesterification with 0.4 M KOH in methanol and analyzed by gas chromatography. The fatty acid content was measured by the normalization method. All measurements were performed in triplicate.

### 3.6. Texture Profile Analysis (TPA)

The adductor muscle part of the oyster was selected for measurement to ensure the uniformity of the sample. The texture meter (TA-XTplus, Stable Micro System, UK), equipped with an aluminum cylindrical probe (diameter P/36–50 mm), was used to tested texture changes of the oyster. The oyster samples were compressed at a compression speed of 5 mm/s to 80% of the initial sample, and the test time was 5 s. Each oyster sample was evaluated by four texture parameters, including hardness, springiness, chewiness, and cohesiveness [50].

### 3.7. Electronic Nose Analysis 

Oyster flavor substances were determined according to previous methods [51]. PEN 3 electronic noses (Winmuster Airsense Analytics Inc., Schwerin, Germany) with 10 kinds of metal oxide semiconductors were used to identify oyster flavor compounds. Briefly, 2.0 g oyster sample was put into a 20 mL airtight bottle and preheated at 50 °C water bath for 50 min. Then, the carrier gas was detected by the electronic nose, which was dry air with a flow rate of 800 mL/min [19]. Electronic nose is a headspace sampling device, which records and analyzes the sensor signals by pattern recognition algorithm when the odor enters the room with a gas sensor array. Different polylines represent different samples by HHP treatment. Fourteen different polylines represent different sensor categories and different volatile component categories. The sensor and the main applications of e-nose are shown in Table S1 (Supplementary Materials).

### 3.8. Determination of Volatile Components

The headspace solid-phase microextraction (HS-SPME) was used to extract the volatile compound components of oysters based on the previous method with slight modification [44]. Briefly, each oyster sample (5.0 g) and 20 µL internal standard (2-octanol, 410 mg/L) were placed in the same 20 mL closed bottle. The headspace bottle was sealed with PTFE silicon and exposed to 60 °C for 30 min. The extracted fiber was exposed to the top of the closed bottle at 40 °C for adsorption and continued for 45 min. Moreover, it was inserted into the GC-MS inlet for desorption at 250 °C for 5 min. The instrument for measuring the volatile components of oyster samples was an Agilent 7890 GC (model 7890A-5975C, Agilent Technologies, Santa Clara, CA, USA), equipped with an HHP-5MS capillary column (30 m × 0.32 mm × 0.25 μm). The temperature adjustment procedure was as follows: the initial temperature was set at 40 °C, then risen to 120 °C at a rate of 3 °C/min, and maintained for 5 min, and then it continued to rise to 200 °C at a rate of 8 °C/min. Pure helium (99.999%) as a carrier gas had a flow rate of 1 mL/min. The ionization energy setting of the detector was set to 70 eV. The ionization energy setting of the detector was set to 70 eV, and the MS transfer temperature was controlled to 250 °C.

### 3.9. Statistical Analysis 

The effect of different HHP treatments (0.1, 100, 200, 300, 400, 500, and 600 MPa) on oyster samples was analyzed by one way of variance. To analyze the effect of HHP treatment on the physical and volatile substances of oyster samples, the mean differences (*p* < 0.05) of all data were obtained through the Turkey test. The iMPact of HHP treatment was evaluated by using ANOVA and SPSS14.0 software (SPSS Inc., Chicago, IL, USA).

## 4. Conclusions

Overall, HHP treatment increased the water content of the oysters, which led to a decrease in the crude protein and ash. HHP also slightly changed the composition of fatty acids. Texture parameters showed that HHP significantly changed the texture of oysters by increasing hardness, springiness, chewiness, and cohesiveness. Moreover, HHP treatment increased the quantity and concentration of volatile components and improved the flavor of raw oysters. The present research results provide a reference for the influence of HHP on oyster flavor and also provide a new idea for the development of aquatic products.

## Figures and Tables

**Table 1:** Effects of HHP treatment on proximate composition and pH of oyster.

	HHP Treatment Pressure (MPa)					
	0.1	100	200	300	400	500
Moisture (%)	82.45 ± 0.23 ^d^	82.35 ± 0.35 ^d^	82.84 ± 0.14 ^c^	83.43 ± 0.22 ^b^	84.65 ± 0.45 ^a^	84.71 ± 0.11 ^a^
Crude protein (%)	12.14 ± 0.25 ^a^	11.48 ± 0.31 ^b^	11.52 ± 0.22 ^b^	11.38 ± 0.21 ^b^	10.54 ± 0.29 ^c^	10.52 ± 0.37 ^c^
Crude lipid (%)	1.41 ± 0.15 ^a^	1.39 ± 0.12 ^a^	1.34 ± 0.26 ^a^	1.38 ± 0.08 ^a^	1.44 ± 0.09 ^a^	1.46 ± 0.12 ^a^
Glycogen (%)	2.61 ± 0.54 ^a^	2.57 ± 0.14 ^a^	2.64 ± 0.57 ^a^	2.59 ± 0.32 ^a^	2.64 ± 0.22 ^a^	2.66 ± 0.23 ^a^
Ash (%)	4.33 ± 0.19 ^a^	4.29 ± 0.16 ^a^	4.14 ± 0.22 ^a^	4.10 ± 0.12 ^a^	3.78 ± 0.16 ^b^	3.64 ± 0.14 ^c^
pH	6.60 ± 0.03 ^b^	6.61 ± 0.01 ^b^	6.63 ± 0.01 ^b^	6.66 ± 0.02 ^a^	6.67 ± 0.02 ^a^	6.66 ± 0.01 ^a^

^a–d^: mean value in the same column with different letters was significantly different (*p* < 0.05) by a Tukey test (*n* = 3).

**Table 2:** Effects of HHP treatment on texture parameters of adductor muscle in oyster.

Pressure (MPa)	Hardness (N)	Springiness (mm)	Chewiness (mJ)	Cohesiveness (Ratio)
0.1	56.68 ± 4.23 ^c^	0.18 ± 0.02 ^d^	2.35 ± 0.53 ^d^	0.22 ± 0.03 ^c^
100	60.06 ± 5.90 ^c^	0.20 ± 0.01 ^d^	2.95 ± 0.33 ^d^	0.24 ± 0.04 ^bc^
200	58.08 ± 7.31 ^c^	0.18 ± 0.02 ^d^	2.43 ± 0.59 ^d^	0.24 ± 0.04 ^bc^
300	60.59 ± 5.99 ^c^	0.26 ± 0.03 ^c^	4.53 ± 1.09 ^c^	0.29 ± 0.02 ^b^
400	101.70 ± 7.53 ^b^	0.32 ± 0.01 ^b^	12.29 ± 1.72 ^b^	0.38 ± 0.04 ^a^
500	114.06 ± 4.07 ^a^	0.39 ± 0.03 ^a^	20.06 ± 2.37 ^a^	0.40 ± 0.02 ^a^

^a–d^: mean value in the same column with different letters was significantly different (*p* < 0.05) by a Tukey test (*n* = 3).

**Table 3:** Fatty acid profiles of oysters treated with different HHP pressure.

HHP Treatment Pressure (MPa)						
Fatty Acid (%)	0.1	100	200	300	400	500
C6:0	1.15 ± 0.33 ^d^	2.11 ± 0.06 ^c^	2.38 ± 0.09 ^b^	2.52 ± 0.15 ^b^	2.86 ± 0.03 ^a^	2.94 ± 0.13 ^a^
C8:0	3.15 ± 0.19 ^a^	1.38 ± 0.17 ^b^	1.45 ± 0.25 ^b^	1.36 ± 0.54 ^b^	1.28 ± 0.37 ^b^	1.31 ± 0.36 ^b^
C10:0	2.27 ± 0.08 ^a^	2.19 ± 0.11 ^a^	1.53 ± 0.12 ^c^	1.59 ± 0.12 ^c^	1.89 ± 0.04 ^b^	1.87 ± 0.03 ^b^
C11:0	2.54 ± 0.11 ^a^	1.85 ± 0.11 ^b^	1.47 ± 0.34 ^b^	1.53 ± 0.24 ^b^	1.76 ± 0.05 ^b^	1.82 ± 0.12 ^b^
C12:0	1.88 ± 0.06 ^b^	2.53 ± 0.07 ^a^	1.55 ± 0.24 ^c^	1.64 ± 0.16 ^c^	1.78 ± 0.05 ^bc^	1.81 ± 0.11 ^bc^
C14:0	2.78 ± 0.22 ^ab^	2.55 ± 0.18 ^b^	2.53 ± 0.19 ^b^	2.62 ± 0.11 ^b^	2.97 ± 0.07 ^a^	2.95 ± 0.08 ^a^
C15:0	2.22 ± 0.09 ^b^	2.19 ± 0.13 ^b^	2.17 ± 0.15 ^b^	2.23 ± 0.06 ^b^	2.59 ± 0.11b ^a^	2.65 ± 0.12 ^a^
C16:0	23.84 ± 0.55 ^a^	24.27 ± 0.96 ^a^	24.78 ± 1.65 ^a^	24.89 ± 1.2 ^a^	25.13 ± 0.68 ^a^	25.33 ± 0.34 ^a^
C17:0	2.26 ± 0.41 ^a^	2.54 ± 0.34 ^b^	2.62 ± 0.44 ^b^	2.73 ± 0.33 ^a^	2.75 ± 0.43 ^a^	2.80 ± 0.34 ^a^
C18:0	11.38 ± 0.87 ^a^	11.88 ± 1.06 ^a^	11.97 ± 0.48 ^a^	11.86 ± 1.27 ^a^	12.33 ± 0.28 ^a^	12.58 ± 1.06 ^a^
C20:0	0.38 ± 0.13 ^b^	0.56 ± 0.13 ^b^	0.97 ± 0.21 ^a^	0.98 ± 0.15 ^a^	1.13 ± 0.22 ^a^	1.15 ± 0.37 ^a^
ΣSFA	54.32 ± 2.13 ^a^	52.70 ± 1.55 ^a^	55.29 ± 2.48 ^a^	53.85 ± 2.39 ^a^	54.55 ± 1.05 ^a^	52.82 ± 2.27 ^a^
C14:1n5	2.32 ± 0.33 ^a^	2.35 ± 0.24 ^a^	2.33 ± 0.21 ^a^	2.31 ± 0.14 ^a^	2.37 ± 0.43 ^a^	2.36 ± 0.52 ^a^
C15:1n9	1.25 ± 0.05 ^a^	1.24 ± 0.12 ^a^	1.23 ± 0.05 ^a^	1.25 ± 0.14 ^a^	1.45 ± 0.15 ^a^	1.47 ± 0.26 ^a^
C16:1n7	2.62 ± 0.27 ^a^	2.55 ± 0.18 ^a^	2.35 ± 0.14 ^a^	2.56 ± 0.23 ^a^	2.93 ± 0.43 ^a^	2.95 ± 0.33 ^a^
C18:1n-7	2.53 ± 0.27 ^a^	2.55 ± 0.06 ^a^	2.47 ± 0.28 ^a^	2.45 ± 0.18 ^a^	2.46 ± 0.12 ^a^	2.38 ± 0.24 ^a^
C18:1n-5	3.75 ± 0.27 ^b^	3.45 ± 0.18 ^b^	3.54 ± 0.23 ^b^	3.58 ± 0.27 ^b^	4.64 ± 0.33 ^a^	4.68 ± 0.69 ^a^
C20:1n9	2.49 ± 0.22 ^a^	2.35 ± 0.17 ^a^	2.38 ± 0.35 ^a^	2.41 ± 0.29 ^a^	2.54 ± 0.12 ^a^	2.62 ± 0.16 ^a^
∑MUFA	13.42 ± 1.34 ^a^	13.67 ± 1.52 ^a^	13.64 ± 1.65 ^a^	14.13 ± 1.35 ^a^	14.03 ± 1.76 ^a^	13.67 ± 1.22 ^a^
C18:2n-6	3.03 ± 0.28 ^a^	2.88 ± 0.11 ^a^	2.52 ± 0.32 ^a^	2.71 ± 0.08 ^a^	2.82 ± 0.22 ^a^	2.88 ± 0.24 ^a^
C18:3n-3	3.14 ± 0.21 ^b^	3.34 ± 0.32 ^b^	3.35 ± 0.11 ^b^	3.34 ± 0.09 ^b^	3.98 ± 0.19 ^a^	4.11 ± 0.17 ^a^
C20:2n6	3.28 ± 0.23 ^a^	3.06 ± 0.27 ^a^	3.08 ± 0.39 ^a^	3.08 ± 0.18 ^a^	3.02 ± 0.36 ^a^	3.05 ± 0.54 ^a^
C20:3n6	2.46 ± 0.29 ^a^	2.41 ± 0.14 ^a^	2.37 ± 0.28 ^a^	2.35 ± 0.17 ^a^	2.24 ± 0.15 ^a^	2.18 ± 0.34 ^a^
C20:4n6 ARA	3.46 ± 0.35 ^a^	3.52 ± 0.21 ^a^	3.34 ± 0.09 ^a^	3.39 ± 0.12 ^a^	3.28 ± 0.15 ^a^	3.14 ± 0.24 ^a^
C20:5n3 EPA	5.35 ± 0.53 ^b^	5.65 ± 0.42 ^b^	5.78 ± 0.13 ^b^	6.13 ± 0.24 ^a^	6.57 ± 0.31 ^a^	6.64 ± 0.25 ^a^
C22:2n6	3.42 ± 0.33 ^b^	3.46 ± 0.47 ^b^	3.46 ± 0.14 ^b^	4.07 ± 0.18 ^a^	4.35 ± 0.05 ^a^	4.54 ± 0.33 ^a^
C22:6n3 DHA	6.10 ± 0.65 ^b^	6.38 ± 0.19 ^b^	6.37 ± 0.35 ^b^	6.42 ± 0.45 ^b^	7.66 ± 0.27 ^a^	7.68 ± 0.25 ^a^
∑PUFA	32.26 ± 3.21 ^a^	30.39 ± 2.59 ^a^	31.07 ± 2.46 ^a^	32.02 ± 1.98 ^a^	32.52 ± 2.13 ^a^	33.51 ± 1.98 ^a^
Σn-3	14.59 ± 1.17 ^b^	15.37 ± 2.35 ^b^	15.50 ± 1.54 ^b^	15.89 ± 0.87 ^b^	18.21 ± 1.16 ^a^	18.43 ± 1.29 ^a^
Σn-6	15.65 ± 1.54 ^a^	15.33 ± 1.37 ^a^	14.77 ± 1.14 ^a^	15.63 ± 1.66 ^a^	15.71 ± 1.35 ^a^	15.79 ± 2.02 ^a^
Σn-3/Σn-6	0.93	0.96	1.06	1.06	1.18	1.21

^a–d^: mean value in the same column with different letters was significantly different (*p* < 0.05) by a Tukey test (*n* = 3).

**Table 4:** Volatile compounds identified by SPME/GC–MS in oysters after HHP treatments.

	Compound	HHP Treatment Pressure (MPa)					
		0.1	100	200	300	400	500
Aldehydes	Hexenoic aldehyde	-	-	-	0.018	0.027	0.040
	Octanal	0.011	-	-	0.015	-	0.015
	2,4-Heptadienal, (E, E)-	0.014	0.010	-	0.017	0.017	0.038
	Benzaldehyde	0.019	-	-	0.017	0.010	0.017
	2-Nonenal, (E)-	0.025	0.019	0.0150	0.061	0.060	-
	2,6-Nonadienal, (E, E)-	0.113	0.162	0.179	0.741	0.630	0.452
	2,4-Octadiene (E, E)	-	-	-	0.013	0.017	0.023
Alcohols	2-Penten-1-ol, (Z)--	-	-	-	-	0.007	0.015
	1-Octen-3-ol	0.333	0.268	0.260	0.388	0.407	0.550
	2-Octen-1-ol, (Z)-	-	-	0.016	-	0.057	0.117
	(6Z)-Nonen-1-ol	0.010	0.015	-	-	-	
	3,6-Nonadien-1-ol, (E, Z)-	0.183	-	0.146	0.088	0.056	0.047
Esters	3-Nonenoic acid-ethyl ester	-	-	0.018	-	-	-
	Tetradecanoic acid-ethyl ester	0.053	0.035	0.041	0.059	0.051	0.045
	Hexadecanoic acid-ethyl ester	0.046	0.029	0.036	0.040	0.036	0.025
	E-11-Hexadecenoic acid-ethyl ester	0.016	0.010	0.009	0.016	0.012	0.011
Ketones	3-Octanone	1.461	0.663	0.644	0.639	0.616	0.648
	2-Undecanone	0.017	0.017	0.015	-	-	-
Hydrocarbon	2-Octene, (E)-	-	-	-	-	-	0.087
	3,5-Octadiene, (Z, Z)-	-	-	-	0.039	0.349	1.183
	1,3-trans,5-cis-octatriene	0.110	0.016	0.101	0.411	0.386	0.438
	E, Z-4-Ethylidenecyclohexene	-	0.028	-	-	-	-
	(E, E, E)-2,4,6-Octatriene	-	-	-	0.033	0.046	0.111
	1,3-cyclooctadiene	0.085	0.131	0.092	0.047	0.437	0.726
	Cyclooctene-3-ethenyl	-	-	-	-	-	0.105
Other class	1,3-Dimethylbenzene	0.022	0.018	0.010	0.016	0.014	0.023
	Benzene, 1-Methyl-3-(1-methylethyl)-	0.006	0.012	-	-	0.017	0.025
	Oxime-, methoxy-phenyl-	0.019	-	0.051	0.034	-	-
	Phenol, 4-ethyl-	-	-	-	-	-	0.014

“-”: Not detected.

## Data Availability

The data presented in this study are available upon request.

## Supplementary Materials

The following are available online, Table S1: Electronic nose sensor and application.

## Sample Availability

Samples of the compounds are available from the authors.
