# Supplementary material for: High Hydrostatic Pressure Treatment of Oysters (Crassostrea gigas)—Impact on Physicochemical Properties, Texture Parameters, and Volatile Flavor Compounds
Source: Molecules. 2021 Sep 22;26(19):5731. doi: 10.3390/molecules26195731 (PMC8510164; doi:10.3390/molecules26195731)
Supplement: Supplementary file 1 [file molecules-26-05731-s001.zip › molecules-1327652-supplementary.pdf]

Supplementary materials

High hydrostatic pressure treatment of oysters (*Crassostrea gigas*) – IMPact on physicochemical properties, texture parameters and volatile flavor compounds

Yuyang Ma[1], Runfang Wang[1], Tietao Zhang[2], Yunsheng Xu[2], Suisui Jiang[1] and Yuanhui Zhao[1*]

[1] College of Food Science and Engineering, Ocean University of China, Qingdao, Shandong 266003, China
[2] College of Food Science and Engineering, Hainan Tropical Ocean University, Sanya, Hainan 572022, China

***Correspondence:** zhaoyuanhui@ouc.edu.cn; Tel./Fax: +86-532-82032783

Supplementary Table.

Table S1. Electronic nose sensor and application

Array number	Sensor name	Performance description
1	S1	Aromatic compounds
2	S2	Nitrogen oxide
3	S3	Triterpenes
4	S4	Organic acid esters
5	S5	Terpenes, esters
6	S6	Sterols
7	S7	Oxygen derivatives of aliphatic hydrocarbons
8	S8	Amines
9	S9	Hydrogen
10	S10	Furans
11	S11	VOC
12	S12	Sulfide
13	S13	Ethylene
14	S14	Lactones, pyrazines
